# Cervical embryonal rhabdomyosarcoma beyond childhood: A case report and literature review

**DOI:** 10.1016/j.radcr.2026.02.029

**Published:** 2026-03-14

**Authors:** Diana Kokash, Zainab Siddiqui, Taahira Arief, Donna Salam, Rashid AlSharhan, Ahmed Al Kindi

**Affiliations:** a Medical Imaging, Dubai Health, Dubai, United Arab Emirates; b Department of Radiology, Graduate Medical Education, Mohammed Bin Rashid University of Medicine and Health Sciences, Dubai Health, Dubai, United Arab Emirates; c Faculty of Medicine, Ivane Javakhishvili Tbilisi State University, Tbilisi, Georgia

**Keywords:** Embryonal rhabdomyosarcoma, Uterine cervix, Botryoid, Cervical cancer

## Abstract

Embryonal rhabdomyosarcoma (ERMS) arising from the uterine cervix is a rare malignancy, predominantly affecting children and adolescents. This case report highlights a rare presentation of cervical ERMS in an adult woman, emphasizing the importance of considering ERMS in the differential diagnosis of a cervical mass in adult women and highlights the critical role of a multidisciplinary treatment approach. A 31-year-old woman presented with acute urinary retention, vaginal bleeding, and postcoital bleeding. Imaging at presentation revealed a large cervical mass exerting a significant mass effect on adjacent structures. Histopathology of the surgically excised mass confirmed the diagnosis of ERMS, with tumor cells arranged in sheets of small, round cells with scant eosinophilic cytoplasm and eccentric, small oval nuclei and inconspicuous nucleoli, supported by immunohistochemical staining positive for desmin, myogenin, and MyoD1. The patient underwent surgical resection; however, she was lost to follow-up, limiting long-term outcome assessment. Cervical ERMS is an aggressive soft tissue sarcoma that rarely occurs in adult women. It can mimic other cervical pathologies, posing diagnostic challenges. Ultrasound and magnetic resonance imaging are key for initial assessment, while early diagnosis, surgery, and adjuvant chemotherapy improve outcomes. Optimal management requires a multidisciplinary team.

## Introduction

Rhabdomyosarcomas are the most common soft tissue tumors in children, accounting for 5% of all pediatric cancers [1]. In contrast, they are extremely rare in adults, representing only 1% of soft tissue sarcomas [2]. These malignant tumors arise from primitive skeletal muscle cells. According to the current WHO classification, rhabdomyosarcomas are categorized into 4 subtypes based on clinicopathological and molecular features: embryonal, alveolar, spindle cell/sclerosing, and pleomorphic [3].

The most common type of rhabdomyosarcoma in early childhood is embryonal, accounting for 70%-80% of rhabdomyosarcomas [1]. Most cases occur in the head and neck region, with the genitourinary system being the second most common site of involvement [4]. While a third of the cases usually manifest in the first 5 years of life, it can also manifest in adulthood [3]. Embryonal rhabdomyosarcoma (ERMS) is further subdivided into spindle cell, botryoid, and anaplastic forms of rhabdomyosarcoma [5].

ERMS may present as a painless cervical or vaginal mass, or with symptoms such as abnormal vaginal bleeding and the classic “grape-like” polypoid mass protruding from the vagina [6]. In some instances, the tumor is detected incidentally during routine gynecological examination, while in others, it may manifest with urinary retention or constipation secondary to compression of adjacent pelvic structures.

The optimal diagnosis and management of rhabdomyosarcoma depend on a multidisciplinary team (MDT) and a multimodal treatment approach, typically involving systemic chemotherapy combined with local control measures such as surgical resection and radiotherapy. However, due to the rarity of this tumor in adult women, there is no universally established treatment protocol, and management is often extrapolated from pediatric guidelines.

## Case presentation

A 31-year-old woman presented to the emergency department with acute urinary retention, vaginal bleeding, and postcoital bleeding. She reported a palpable pelvic mass. She denies any past medical, surgical history, or family history of cancer. Vaginal speculum examination revealed a large, friable cervical mass.

Labs revealed that she was anemic with hemoglobin measuring 7.3 g/dL. Tumor marker serology, including CA-125, CA-19-9, carcinoembryonic antigen (CEA), and alpha-fetoprotein (AFP), was all within normal limits. Human papilloma virus (HPV) testing was negative.

A pelvic examination under anesthesia confirmed that the mass was protruding against the posterior vaginal wall and obscuring both ureteral orifices.

**Fig. 1:**
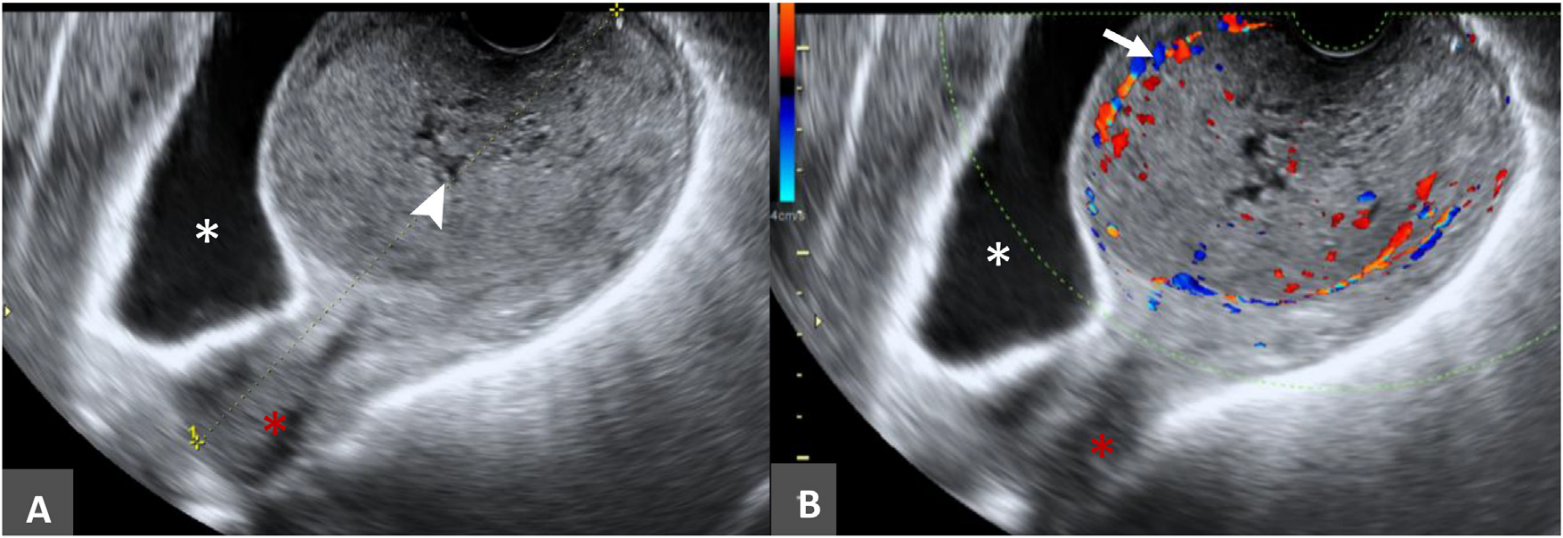
Initial imaging at presentation. (A) Transvaginal grayscale ultrasound: well-circumscribed, heterogeneous mass within the cervical canal with internal cystic spaces. (B) Color Doppler: peripheral vascularity surrounding the lesion.

Transvaginal ultrasound performed at presentation showed a well-circumscribed, heterogeneous large mass demonstrating peripheral vascularity within the cervical canal and showing internal cystic spaces (Fig. 1).

**Fig. 2:**
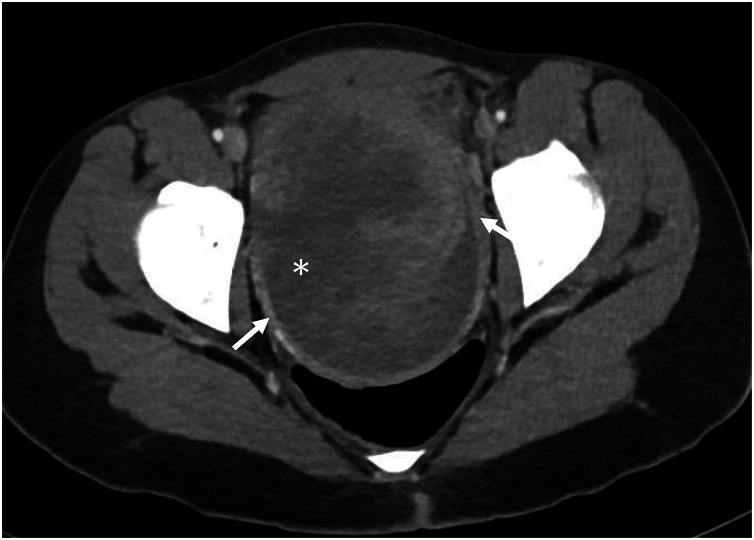
Contrast-enhanced axial computed tomography (CT): large peripherally enhancing (arrows) heterogeneous cervical mass with central fluid attenuation representing hemorrhagic component (asterisk).

Computed tomography (CT) of the abdomen and pelvis performed for further staging demonstrated a large peripherally enhancing mass containing a soft tissue component and central hypodense areas consistent with necrosis. The lesion measured 10 × 13 × 16 cm^3^ (anteroposterior × transverse × craniocaudal) and exerted significant mass effect, displacing the uterus and right ovary superiorly and compressing the urinary bladder and distal left ureter, resulting in left-sided hydronephrosis. Multiple enlarged, conglomerated lymph nodes were seen in the left pelvis and lower left para-aortic region, with the largest measuring up to 6 × 2.6 cm^2^. No evidence of distant metastasis. Based on CT findings, the case was staged as FIGO stage IIIC2 (Fig. 2).

**Fig. 3:**
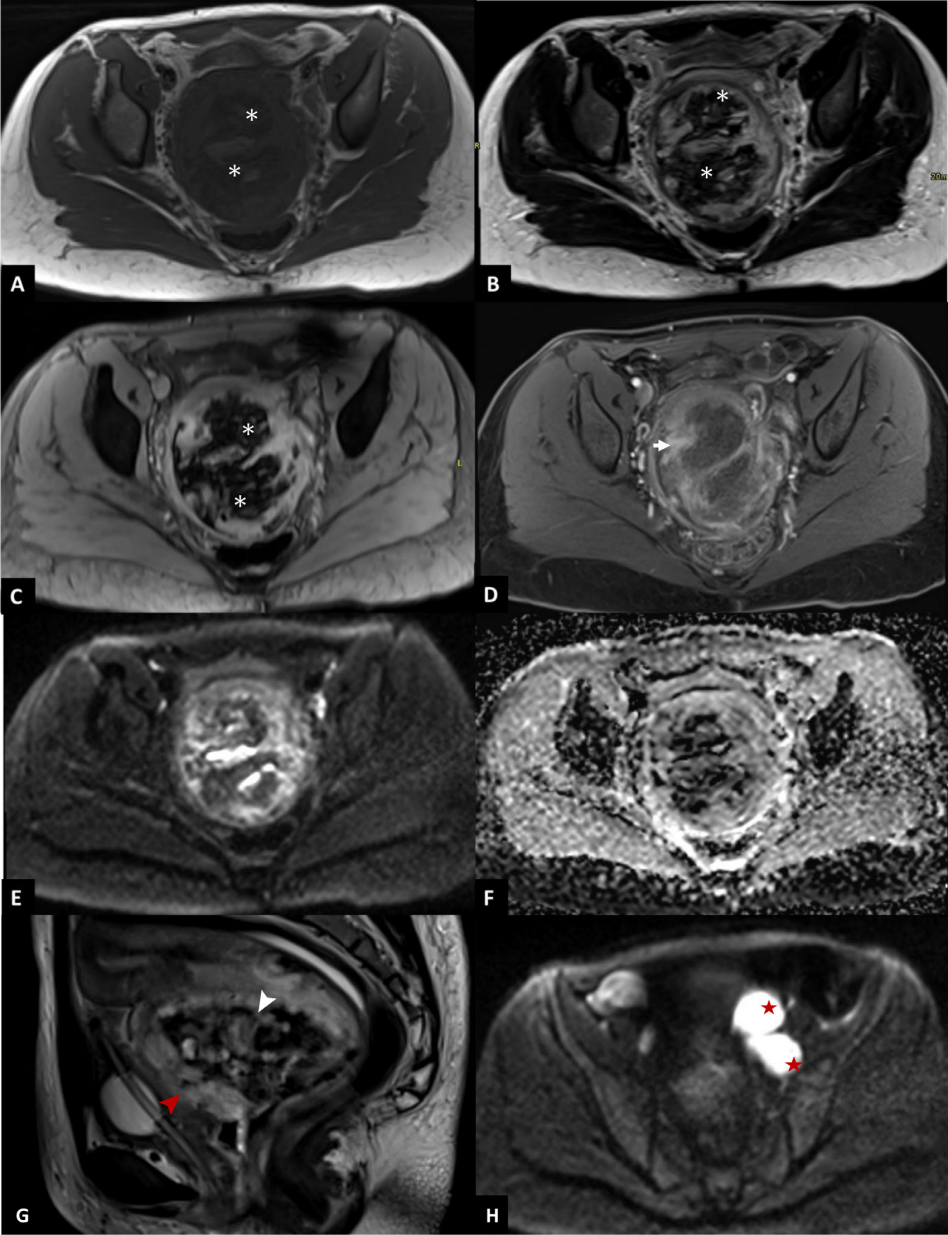
Magnetic resonance imaging (MRI) findings. (A) T1-TSE axial oblique: heterogeneous cervical mass predominantly isointense to muscle with an irregular hypointense necrotic component (asterisks). (B) T2-TSE axial oblique: heterogeneous signal with T2-hyperintense necrotic areas and the same irregular hypointense component (asterisks). (C) GRE T2 axial oblique: blooming of the hypointense component (asterisks), consistent with hemorrhagic products. (D) T1-FS axial oblique post-contrast images show peripheral enhancement (arrows). (E) DWI *b* = 400: minimal diffusion restriction within the solid peripheral component. (F) ADC map: corresponding hypointensity on ADC. (G) T2 sagittal: shows craniocaudal extension with invasion of the uterine isthmus (white arrowhead), uterine body, and posterior bladder wall (red arrowhead), with effacement of the fat plane to the rectum. (H) DWI: enlarged left pelvic lymph nodes (stars).

Magnetic resonance imaging (MRI) of the pelvis, performed for further lesion characterization, demonstrated a heterogeneous mass with a peripheral enhancing component that was isointense to muscle on T1-weighted imaging and showed minimal restricted diffusion. The lesion contained a uniformly hypointense component across all sequences, with blooming artifact on susceptibility-weighted imaging, consistent with hemorrhagic content. Additionally, a T2-hyperintense component with no enhancement in the postcontrast sequences corresponded to necrotic regions. The mass infiltrated the uterine isthmus and part of the uterine body, as well as the vaginal walls, posterior bladder wall, and urethra, with obliteration of the intervening fat planes between the mass and the rectum (Fig. 3).

MDT discussion recommended total abdominal hysterectomy with bilateral salpingectomy and left pelvic lymphadenectomy, which the patient underwent. The patient was referred to another facility to continue care at a dedicated oncology unit.

**Fig. 4:**
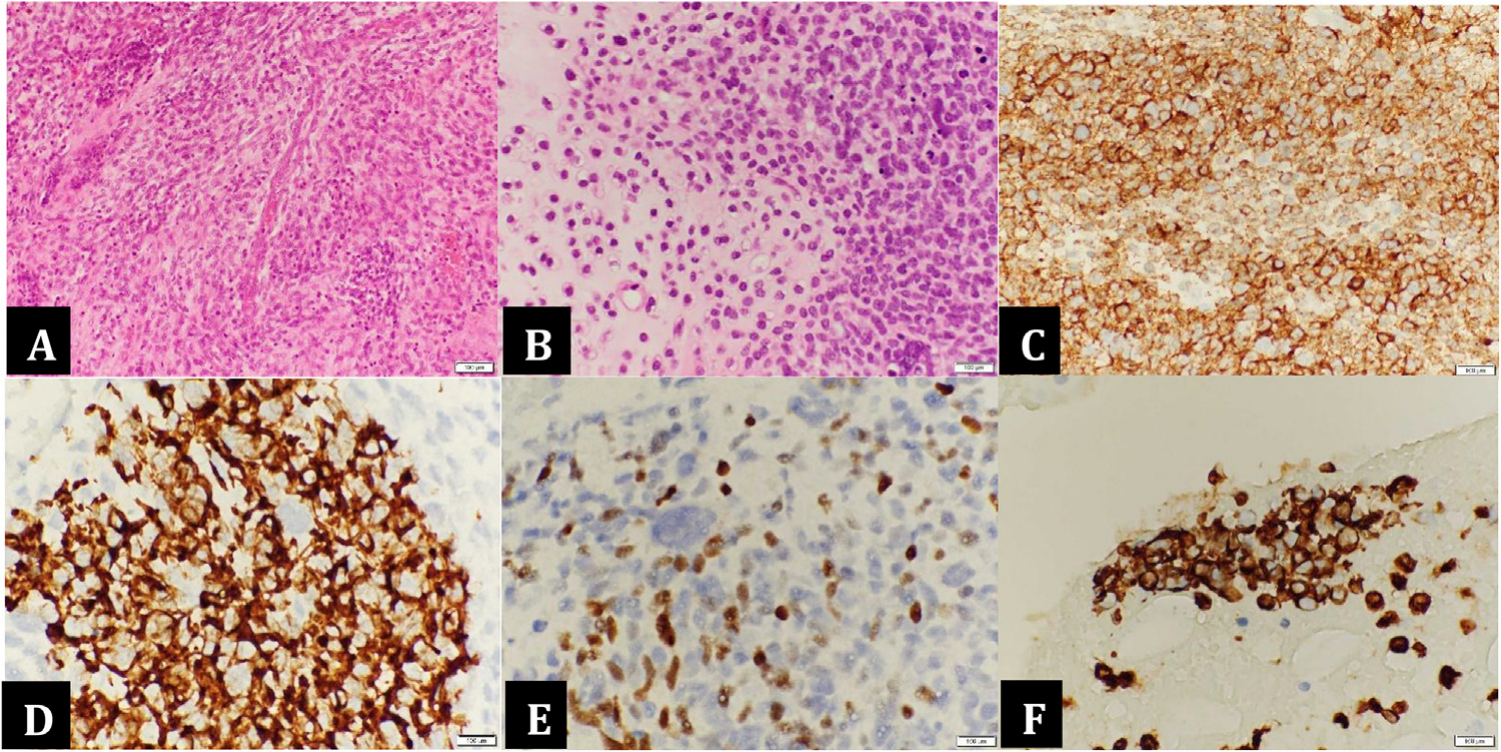
Histopathology and immunohistochemistry: (A) High-power view (×40) showing sheets of neoplastic small round blue cells. (B) Intermediate-power view (×20) demonstrating sheets of neoplastic small round blue cells with areas of cartilaginous differentiation. (C-F) Immunostaining with high-power view (×60) was positive for caldesmon (C), desmin (D), myogenin (E), and vimentin (F).

Histopathological examination of the excised specimen revealed a tumor composed of primitive mesenchymal cells arranged in sheets, with hypocellular and hypercellular areas in a myxoid stroma. The tumor cells had scant eosinophilic cytoplasm, eccentric oval nuclei, and inconspicuous nucleoli. Immunohistochemistry demonstrated positive staining for S100, highlighting the cartilaginous component. The non-cartilaginous components were positive for desmin, p63, caldesmon, CD10, myogenin, and CD117. Immunomarkers, such as CK AE1/AE3, IHD1, CD99, and synaptophysin, were negative. In an extended immunohistochemistry panel, myogenin, CD10, and CD117 showed focal staining in the undifferentiated areas, while markers, such as Cyclin D1, SMA, and ER, were negative. Intermixed areas of cartilaginous differentiation supported the diagnosis of ERMS with extensive cartilaginous differentiation, a pattern commonly associated with DICER1 syndrome-related tumors (Fig. 4).

## Discussion

### Overview

ERMS is an aggressive mesenchymal tumor that arises from immature striated skeletal myocytes. Cervical ERMS is exceptionally rare, accounting for 0.4%-1.0% of all cervical cancer cases. It primarily affects the pediatric population and adolescents, with the mean age of incidence being approximately less than 25 years, and is rarely seen in adult women [7].

The exact pathogenesis for the development of ERMS of the cervix is not fully understood. However, studies have suggested a link to inherited mutations in the DICER1 gene, which encodes an endoribonuclease involved in microRNA production and regulation of protein synthesis [8].

Patients often present with nonspecific clinical symptoms such as painless vaginal bleeding or an exophytic cervical mass, which can be clinically mistaken for a cervical polyp or leiomyoma. In the present case, the patient presented with urinary retention due to mass effect exerted by the tumor [8].

### Imaging features

Imaging plays an important role in delineating tumoral extent and aiding in preoperative surgical planning. It also plays an important role in the follow-up of patients post-resection for detecting recurrence and for monitoring disease progression [2]. However, final diagnosis can be made only on histopathology.

#### Ultrasound

Ultrasound may be utilized in the initial assessment of patients; however, the reported findings are nonspecific and can include a hyperechoic mass with peripheral vascularity [9].

#### Computed tomography

CT is primarily used for staging and detecting distant metastasis. Imaging findings are not specific, typically showing a heterogeneously enhancing mass due to regions of necrosis. Overall, the imaging features resemble rhabdomyosarcomas arising from other anatomical sites.

#### Magnetic resonance imaging

MRI is the preferred modality of choice for local tumor assessment. On MRI, the lesions typically show intermediate to low signal intensity on T1-weighted sequences and high signal intensity on T2-weighted sequences, which represent the necrotic component. Post-contrast imaging often reveals heterogeneous enhancement, suggestive of intralesional necrosis, particularly in larger tumors [10].

In a retrospective review of imaging findings in adult rhabdomyosarcomas, Steven D. Allen et al. reported that lymphadenopathy was present in the majority of patients at the time of imaging—a finding less commonly seen in the pediatric population. Additionally, most tumors in their study demonstrated intralesional necrosis. Both these findings were also observed in our case [2].

### Differential diagnosis

A cervical leiomyoma was considered less likely in our case because there was pelvic and retroperitoneal lymphadenopathy. On imaging, leiomyomas are typically hypoechoic on ultrasound, show predominantly low T2-weighted signal, and demonstrate homogeneous post-contrast enhancement. An additional consideration is cervical leiomyosarcoma, which, unlike our patient, more often presents in perimenopausal or postmenopausal women. It usually appears as a large, heterogeneous mass with intratumoral hemorrhage and necrosis, is typically solitary, and has a thick, enhancing wall. These features help distinguish it from degenerating leiomyomas, which are often multiple and may display a thin T2-hypointense rim due to methemoglobin deposition [11].

Other malignant cervical tumors, including adenocarcinoma and squamous cell carcinoma, can share imaging features with rhabdomyosarcoma. As tumor size increases, imaging specificity declines, and histopathologic confirmation is often required [12].

Benign mimics, such as endocervical polyps and nabothian/lobular endocervical glandular hyperplasia multicystic lesions, typically show a multicystic, high signal on T2-WI with thin septa, smooth noninvasive margins, and no aggressive diffusion restriction [13].

### Histopathology

Histopathology of ERMS is typically characterized by sheets of primitive mesenchymal cells, consisting of small round and spindle-shaped cells with hyperchromatic nuclei, scant eosinophilic cytoplasm, and a myxoid stroma. As the tumor cells undergo differentiation, they develop more eosinophilic cytoplasm and form elongated shapes, commonly seen post-chemotherapy. Immunohistochemically, skeletal muscle differentiation markers are positive, showing diffuse staining of Desmin with patchy Myogenin and MYO-D1 [5].

### Management and treatment strategies

Cervical ERMS is managed with multimodal therapy in a MDT. Induction multi-agent chemotherapy, commonly VAC (vincristine, actinomycin D, cyclophosphamide) or IVA (ifosfamide, vincristine, actinomycin D), is typically used to downstage the tumor, followed by organ-preserving surgery when feasible. Re-excision is considered for positive margins. Radiotherapy is used selectively for residual or margin-positive disease, nodal involvement, or non-resected lesions that do not achieve complete response with chemotherapy. When radiotherapy is indicated for cervix or vagina, image-guided brachytherapy is preferred to optimize local control while limiting late effects. Favorable chemotherapy response in vaginal or uterine primary lesions often negates the need for radical surgery, prompting emphasis on fertility preservation, late-effect reduction, and close surveillance. Response assessment with interval imaging and exams guides the timing and extent of local therapy, and regional nodal evaluation is individualized based on imaging findings [14,15].

## Conclusion

Cervical ERMS is extremely rare in adult women and can mimic other cervical neoplasms, leading to diagnostic challenges. Early recognition, prompt surgical intervention, and adjuvant chemotherapy are essential for improving survival outcomes. A multidisciplinary approach involving gynecologic oncologists, pathologists, and radiologists is vital for optimizing management and long-term prognosis.

## Patient consent

Written informed consent was obtained from the patient for publication of this case report and any accompanying images.

## References

[1] Skapek SX, Ferrari A, Gupta AA, Lupo PJ, Butler E, Shipley J (2019). Rhabdomyosarcoma. Nat Rev Dis Primers.

[2] Allen SD, Moskovic EC, Fisher C, Thomas JM (2007). Adult rhabdomyosarcoma: Cross-sectional imaging findings including histopathologic correlation. AJR Am J Roentgenol.

[3] Dehner CA, Rudzinski ER, Davis JL (2024). Rhabdomyosarcoma: Updates on classification and the necessity of molecular testing beyond immunohistochemistry. Hum Pathol.

[4] Zeisler H, Mayerhofer K, Joura EA (1998). Embryonal rhabdomyosarcoma of the uterine cervix: Case report and review of the literature. Gynecol Oncol.

[5] Agaram N.P. (2022). Evolving classification of rhabdomyosarcoma. Histopathology.

[6] Kriseman M.L., Wang W-L, Sullinger J., Schmeler K.M., Ramirez P.T., Herzog C.E. (2012). Rhabdomyosarcoma of the cervix in adult women and younger patients. Gynecol Oncol.

[7] Ibrahim U, Saqib A, Mohammad F, Ding J, Salman B, Collado FK (2017). Embryonal Rhabdomyosarcoma of the Cervix: A Rare Disease at an Uncommon Age. Cureus.

[8] Bahall V., De Barry L., Sankar S. (2022). A rare case of embryonal rhabdomyosarcoma of the uterine cervix. Case Rep Pathol.

[9] AlMulai I., Fahad A., Nagshabandi Z. (2025). Cervical rhabdomyosarcoma: a case report. Cureus.

[10] Espinoza J.L.H., Figueroa JVJE, Díaz V.E.V., Amesquita L.S.H., Baca R.C.C., Vega M.R. (2024). Soft-tissue sarcomas of the genitourinary tract with radiologic-pathologic correlation. Radiographics.

[11] Hindman N., Kang S., Fournier L., Lakhman Y., Nougaret S., Reinhold C. (2023). MRI evaluation of uterine masses for risk of leiomyosarcoma: a consensus statement. Radiology.

[12] Mansoori B., Khatri G., Rivera-Colón G., Albuquerque K., Lea J., Pinho D.F. (2020). Multimodality imaging of uterine cervical malignancies. Am J Roentgenol.

[13] Saida T, Sakata A, Tanaka YO, Ochi H, Ishiguro T, Sakai M (2019). Clinical and MRI Characteristics of Uterine Cervical Adenocarcinoma: Its Variants and Mimics. Korean J Radiol.

[14] Lautz T.B., Martelli H., Fuchs J., Chargari C., Smeulders N., Granberg C (2020).

[15] Minard-Colin V, Walterhouse D, Bisogno G, Martelli H, Anderson J, Rodeberg DA (2018). Localized vaginal/uterine rhabdomyosarcoma-results of a pooled analysis from four international cooperative groups. Pediatr Blood Cancer.

